# Property and Stability of Astaxanthin Emulsion Based on Pickering Emulsion Templating with Zein and Sodium Alginate as Stabilizer

**DOI:** 10.3390/ijms23169386

**Published:** 2022-08-20

**Authors:** Yan Xu, Zhe Jia, Jiaxing Wang, Jipeng Sun, Ru Song

**Affiliations:** 1 Key Laboratory of Health Risk Factors for Seafood of Zhejiang Province, School of Food Science and Pharmacy, Zhejiang Ocean University, Zhoushan 316022, China; 2 Research Office of Marine Biological Resources Utilization and Development, Zhejiang Marine Development Research Institute, Zhoushan 316021, China

**Keywords:** astaxanthin, Pickering emulsion, zein/sodium alginate complex, structure characterization, stability, degradation kinetics

## Abstract

Astaxanthin loaded Pickering emulsion with zein/sodium alginate (SA) as a stabilizer (named as APEs) was developed, and its structure and stability were characterized. The encapsulation efficiency of astaxanthin (Asta) in APEs was up to 86.7 ± 3.8%, with a mean particle size of 4.763 μm. Freeze-dried APEs showed particles stacked together under scanning electronic microscope; whereas dispersed spherical nanoparticles were observed in APEs dilution under transmission electron microscope images. Confocal laser scanning microscope images indicated that zein particles loaded with Asta were aggregated with SA coating. X-ray diffraction patterns and Fourier transform infrared spectra results showed that intermolecular hydrogen bonding, electrostatic attraction and hydrophobic effect were involved in APEs formation. APEs demonstrated non-Newtonian shear-thinning behavior and fit well to the Cross model. Compared to bare Asta extract, APEs maintained high Asta retention and antioxidant activity when heated from 50 to 10 °C. APEs showed different stability at pH (3.0–11.0) and Na^+^, K^+^, Ca^2+^, Cu^2+^ and Fe^2+^ conditions by visual, zeta potential and polydispersity index measurements. Additionally, the first order kinetics fit well to describe APEs degradation at pH 3.0 to 9.0, Na^+^, and K^+^ conditions. Our results suggest the potential application of Asta-loaded Pickering emulsion in food systems as a fortified additive.

## 1. Introduction

As the people of the world become more aware of health, consumers pay more and more attention to the safety, nutrition and bioavailability of food they intake. In particular, there is a great interest in the development of functional foods fortified with one or more health-promoting ingredients, such as vitamins, minerals, or natural nutraceuticals [1]. Among these bioactive ingredients, astaxanthin (3, 3′-dihydroxy-β, β′-carotene-4, 4′-dione, Asta) is famous for its strong antioxidant activity, with 10 times the antioxidant activity of β-carotene and lutein, and is about 100 times more effective than α-tocopherol [2,3]. In addition, Asta can reduce cardiovascular disease and improve anti-cancer and immune systems [4,5]. However, natural Asta is sensitive to environmental factors, such as light, heat, and ionic strength, etc., which will lead to Asta degradation, undesirable product generation and functional property loss [6]. The strong hydrophobicity of Asta also determines its low bio-accessibility in an aqueous solution. The above limitations of natural Asta will limit its wide application in food or other industries.

An emulsion-based system is one of the most attractive carriers for environment sensitive or weak bio-accessibility components by constructing different emulsion structures to regulate the release of active compounds under different conditions [7]. In recent years, Pickering emulsion has attracted great attention as a drug delivery system, in which food grade surface active colloidal particles are used as stabilizers instead of traditional surfactants (e.g., Twain 80) [8]. Pickering emulsions stabilized by polysaccharide and/or protein-based micro/nanoparticles have many advantages, such as high stability, good biocompatibility, friendly to environment, etc. [9,10,11].

As a safe food grade material recognized by Food and Drug Administration (FDA), zein is commonly used as a delivery carrier for hydrophobic active compounds due to its high hydrophobicity [12]. In Pickering emulsion, zein can fabricate colloidal particles by anti-solvent precipitation [13]. The addition of polysaccharides into zein-stabilized emulsion to form a protective structure will enhance the stability of hydrophobic compounds encapsulated in Pickering emulsions. For example, the antioxidant Pickering emulsion stabilized with zein–chitosan composite particles remained stable after 270 days storage under ambient temperature [14]. The bio-accessibilities of hydrophobic nutraceuticals using Pickering emulsion as a template were dramatically increased, such as curcumin fabricated with zein/carboxymethyl dextrin nanoparticles [15], curcumin and resveratrol in double-cross-linked emulsion gels with zein and sodium alginate (SA) as mixed biopolymers [16].

To our knowledge, the information of Asta-loaded Pickering emulsion stabilized with zein (protein) and SA (polysaccharide) as composite stabilizer is limited. In this study, a Pickering emulsion was developed with zein and SA as surface-active colloidal particles to encapsulate hydrophobic Asta extract derived from Penaeus sinensis by-products. The property of Asta-loaded zein/SA Pickering emulsion (named as APEs) was measured, and the intermolecular interaction of APEs was analyzed. For further potential application of APEs, we focused on its stability under heating, different pH and metal ions treatments. In addition, the degradation kinetics of Asta in APEs at different pH buffers and metal ions solutions were described or discussed. Our results will provide valuable information for preparation of Asta-loaded Pickering emulsion stabilized with zein/SA complex and suggest its potential application conditions.

## 2. Results and Discussion

### 2.1. Appearance, Particle Size and Morphological Property

**Figure 1:**
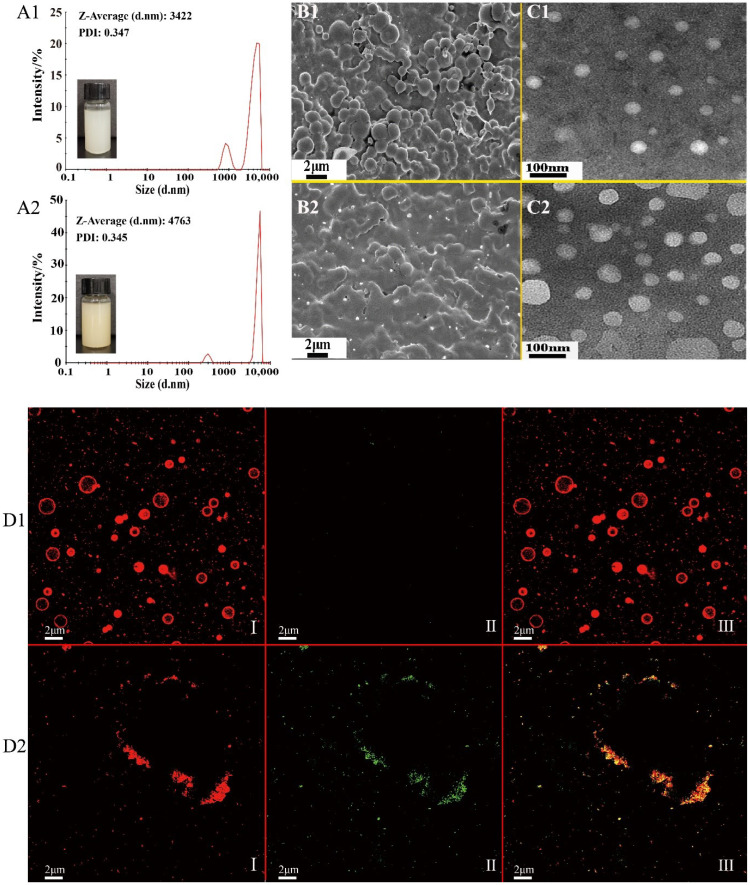
Changes of visual appearance and morphological property of APEs compared with PEs. A—appearance and particle size distribution of PEs (**A1**) and APEs (**A2**), B—SEM images of PEs (**B1**) and APEs (**B2**), C—TEM images of PEs (**C1**) and APEs (**C2**), and D—CLSM images of PEs (**D1**) and APEs (**D2**). In (**D1**) and (**D2**), “I, II, and III” represented zein stained red with fast green FCF, Asta oil phase stained green with Nile Red, and double channel images formed by I and II, respectively.

The appearances of PEs (Pickering emulsion stabilized with zein/SA and no Asta loading) and APEs were uniform and stable. Further, a slight red color was observed for APEs due to Asta being entrapped (Figure 1A1,A2). PEs showed a uniform distribution with an average particle size of 3.422 μm, with a polydispersity index (PDI) of 0.347. By comparison, the average particle size of APEs increased to 4.763 μm after Asta loading; whereas its PDI (0.345) value was similar to that of PEs. APEs are soluble in water at any ratio, and the encapsulation efficiency of Asta in APEs was 86.7 ± 3.8% with 0.420 μg/mg of Asta.

Under scanning electronic microscope (SEM) observation, PE powders were in spherical shapes with smooth surfaces (Figure 1B1). Compared with PEs, freeze-dried APEs had no obvious boundary, but were connected together, as was shown in Figure 1B2. It was noted that irregular microparticles were also observed in the SEM images of PEs and APEs, which might be ascribed to the drying method used. In the lyophilization process, the water removal under vacuum could result in particles accumulation [17]. A similar phenomenon was observed for double-cross-linked emulsion gels using zein and sodium alginate [16].

After being diluted with distilled water, most of the particles in PEs and APEs were spherical nanoparticles in the transmission electron microscope (TEM) images (Figure 1C1,C2), and no obvious agglomeration phenomena were observed, suggesting the obtained APEs and PEs showed good dispersibility in water. The size of zein particles, white oil droplets under negative staining in TEM images, was in accordance with zein nanoparticles (100–200 nm) formed using the antisolvent precipitation method [18].

Confocal laser scanning microscope (CLSM) is usually used to observe the distribution of oil droplets and proteins in emulsions. In a typical CLSM diagram, the part with red color represents protein stained by fast green, while the green color represents the oil droplets stained by Nile red [19]. As shown in Figure 1D1, PEs, without Asta loading, contained a relatively uniform dispersion of spherical complex of zein. After addition of Asta, the images of Figure 1D2(I–III) indicated the oil phase of Asta were entrapped in zein particles. In addition, the existence of aggregates in APEs was observed in CLSM images, which should be due to the cross-linking of zein particles with SA coating.

### 2.2. Molecular Interaction

**Figure 2:**
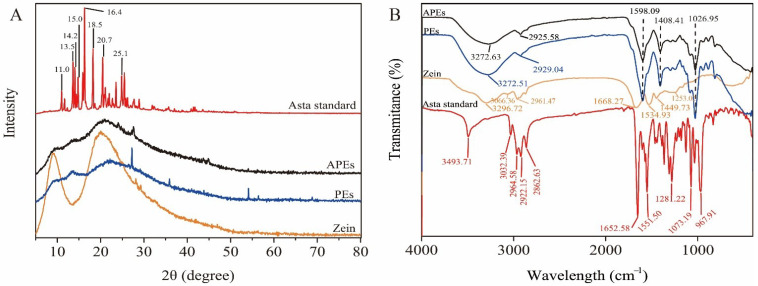
X-ray diffraction (XRD) and Fourier transform infrared spectroscopy (FTIR) spectra of APEs compared with PEs, zein and Asta standard. (**A**) XRD analysis in the range of 5–90°, and (**B**) FTIR analysis from 400 to 4000 cm^−1^.

Asta standard showed strong characteristic peaks at 11.0°, 13.5°, 14.2°, 15.0°, 16.4°, 18.5°, 20.7°, and 25.1° (Figure 2A), suggesting high degree of crystallinity [20]. Consistent with previous reports [19,21], the diffraction peaks of zein powder at 9.2° and 19.7° were also detected in this study. After interaction with SA, the peaks at 9.2° and 19.7° shifted into 13.4° and 22.3° in PEs, respectively. In addition, new peaks were found at 27.3°, 35.9° and 54.1° in the 2θ diffractogram of PEs. These changes reflected the modification in the arrangement of molecules between SA and zein by ionic interaction [22]. After encapsulation of Asta into PEs, the peak intensity at 27.2° decreased, as well as the peaks at 35.9° and 54.1° disappeared in APEs. Furthermore, the peak of 13.4° shifted into 14.2° and the peak intensity around 21° further increased after Asta loaded in PEs. These changes of diffraction angles in APEs indicated the reduction of ionic interaction between zein and SA to a certain extent due to a hydrophobic Asta addition.

The changes of spectral position of the characteristic bands can be reflected in FTIR, which contributes to monitor the intermolecular interactions among the components of complexes [23,24]. The results of Figure 2B showed that Asta standard had characteristic bands at 3493.71 cm^−1^ (–OH), 3032.39 cm^−1^ (=C–H stretching), 3000–2800 cm^−1^ (C–H stretching), 1073.19 cm^−1^ (telescopic vibration of C–O), and 967.91 cm^−1^ (C=C stretching). Furthermore, the characteristic bands including 1652.58 cm^−1^ assigned to C=O stretching vibration band of the six-membered ring and 1551.50 cm^−1^ related to its conjugated structure [25], were detected in Asta standard. As for zein, the spectra of characteristic bands were in accordance with previous studies, such as 3296.72 cm^−1^ assigned to the amide A (NH–stretching coupled with hydrogen bonding), 3066.36 and 2961.47 cm^−1^ assigned to the amide B (antisymmetric and symmetric stretching of C–H), 1668.27, 1534.93, and 1253.02 cm^−1^ associated with the corresponding amide I (C–O stretching vibrations), amide II (C–N stretching vibrations and N–H bending), and amide III (N–H and C–N in-plane bending of bound amide or –CH_2_ groups of glycine) [26,27].

Compared with zein, almost all of characteristic peaks in PEs occurred in shifts. For example, the peak at 3272.51 cm^−1^ shifted from 3296.72 cm^−1^ attributed to the stretching vibration of the O–H in hydrogen bonding [28], and the amide I band at 1598.09 cm^−1^ shifted from 1668.27 cm^−1^ associated with the C=O stretching vibrations in electrostatic interaction [26]. Obviously, these shifts of characteristic bands suggested that PEs was fabricated by hydrogen bonding and electrostatic interaction between zein and SA. However, APEs showed a similar spectrum to PEs, indicating that Asta was successfully embedded in PEs. Hydrophobic interaction should be an important driving force involved in APEs formation due to the hydrophobic properties of Asta and zein. As expected, the peak band at 2929.04 cm^−1^ in PEs was red-shifted to 2925.58 cm^−1^ in APEs, proving C–H antisymmetric stretch mode associated with hydrophobic interaction induced by Asta incorporation. Similar results were found in our previous studies [29]. The findings of Figure 2 suggested that hydrogen bonding, electrostatic attraction and hydrophobic effect were involved in the intermolecular action of Asta loaded Pickering emulsion stabilized by zein/SA.

### 2.3. Rheological Property

The rheological properties of emulsion samples, such as apparent viscosity (*η*), storage modulus (*G′*), and the loss modulus (*G′′*), could provide important information for emulsion stability and droplet–droplet interactions, and therefore suggest their potential applications [30,31].

**Figure 3:**
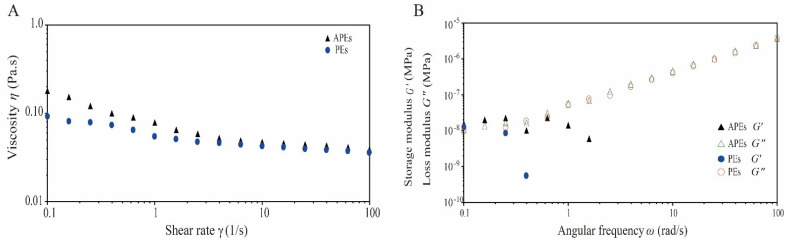
Rheological properties of APEs as compared with PEs at 25 °C. (**A**) Viscosity (*η*) as a function of shear rate (*γ*) increased from 0.1 to 100 s^−^^1^, (**B**) dynamical oscillatory frequency sweep test curves of storage modulus (*G′*) and loss modulus (*G′′*) vs. angular frequency (*ω*) from 0.1 to 100 rad/s.

In this study, the apparent viscosity of APEs gradually decreased as the shear rate increased from 0.1 to 100 s^−1^ (Figure 3A). A similar phenomenon was found in PEs. In pseudoplastic fluids, shear thinning behavior is a typical phenomenon because the crimped and entangled molecular structures are straightened and reoriented at high shear rates [32]. Obviously, the APEs and PEs belonged to pseudoplastic fluids. The destruction of the SA layer outside PEs and APEs might be the main reason for the decreased viscosity with the shear rate increases. However, the APEs demonstrated higher viscosity values than PEs within the shear rate range measurement. This was presumably ascribed to the decrease of separation distance and the increase of interaction between droplets in APEs due to the encapsulation of hydrophobic Asta in PEs, which contributed to the formation of aggregates (as shown in Figure 1D2), so that APEs could resist shear to a certain extent and showed higher initial viscosity.

As shown in Table 1, the zero-shear viscosity (*η*0), the infinite shear viscosity (*η∞*), and the characteristic time (*α*) were found to increase in APEs as compared to those in PEs. The rate index (*n*) was closed to 2/3, which proved that PEs and APEs were water-soluble polymers. The apparent viscosity data of APEs and PEs fit well to the Cross model (*R*^2^ > 0.98) (Table 1), suggesting their shear thinning properties. Similar to our results, Ma, Lin, Chen, Zhao and Zhang (2014) reported that lower concentrations of SA solutions (1.0–3.0%, *w*/*v*) exhibited non-Newtonian shear-thinning behavior and the flow curves could be well described by the Cross model [33]. In this study, the final concentration of SA interacting with zein particles in the APEs formation system (see Section 2.2) was 1.0 wt%, belonged to the low concentration range of SA solution, which might be an important factor leading to the pseudoplastic fluid behavior of APEs.

The viscoelastic behaviors of APEs and PEs at angular frequencies ranging from 100 to 0.1 rad/s were evaluated by undertaking dynamic oscillatory measurements. As was shown in Figure 3B, the storage modulus (*G′*) and loss modulus (*G′′*) of APEs were similar at low frequencies. Similar results were found for the PEs. At lower angular frequency, the polymer chains of SA could have more time to relax to a more favorable state through the slip of the entanglement point of SA chains [33], thereby the majority of energy generated during lower deformation rate could be dissipated by viscous flow [34]. This could explain the closer *G′* and *G′′* values in APEs or PEs observed at a lower angular frequency. The *G′′* values were found to increase for APEs or PEs when the angular frequency increased; whereas decreased *G′* values were detected with the increase of angular frequency for APEs or PEs. At higher angular frequencies, the dynamic mechanical loss tangent (*G′′/G′*) of APEs or PEs aqueous solutions increased dramatically (>1), indicating its predominant viscous behavior. In addition, the tendency of *G′′* values of APEs and PEs to approach each other at high frequency suggested the existence of similar microstructures.

### 2.4. Thermal Stability

**Figure 4:**
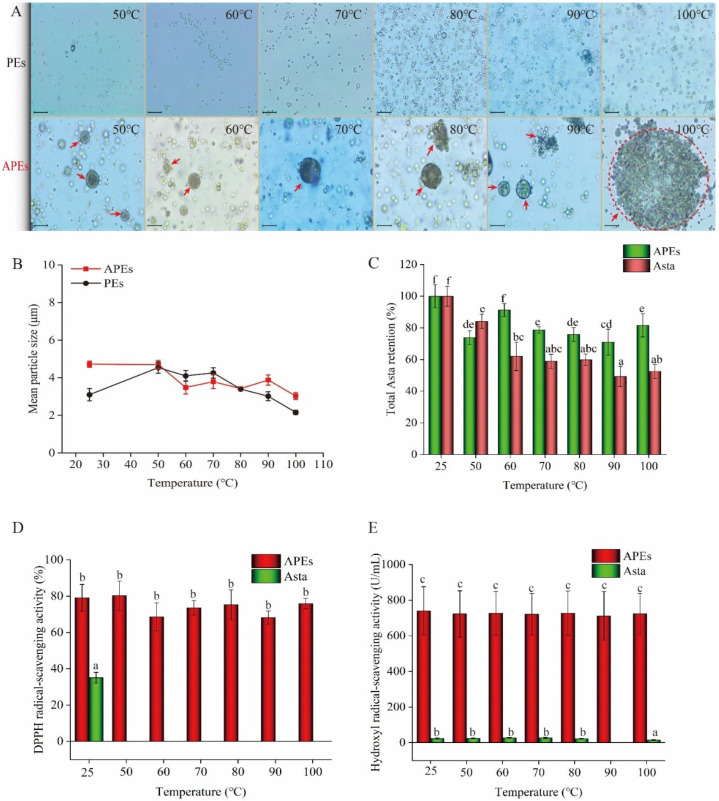
Thermal stability of APEs after 30 min of treatment at 25 °C to 100 °C. PEs or bare Asta was used as comparison. (**A**) optical micrograph changes of APEs, scale at 400 times magnification, (**B**) mean particle size of APEs, (**C**) total Asta retention rate in APEs as compared to bare Asta, (**D**) DPPH radical-scavenging activity of APEs compared with bare Asta, and (**E**) hydroxyl radical-scavenging activity of APEs compared with bare Asta. Data were expressed as the mean ± standard deviation (*n* = 3). Different lower-case letters on the bars in (**C**–**E**) indicate significant differences (*p* < 0.05).

Smaller droplets were observed to aggregate in APEs with temperature increases (Figure 4A). The increased Brownian motion of particles under high temperature could lead to redistributing particles on the oil/water interface and exposing the surfaces of the droplets, which might increase the coalesce of the formed smaller droplets [35]. Similar to our results, Keerati-u-rai and Corredig (2009) described that soy protein isolates emulsions had smaller oil droplet sizes after being heated at 75 °C and 95 °C; however, some cross-linking bridges existed between the droplets [36].

The addition of hydrophobic Asta in PEs system contributed to adsorb more zein molecules and to form a denser cross-linking structure with SA (as shown in Figure 1B2), thus resulting in large particle size of APEs as compared to PEs (Figure 4B). Under heat treatment, decreased mean particle size of APEs should be related to the cracking of large particles into small particles to further stabilize the emulsion. When the thermal temperature exceeded 80 °C, the mean particle size of PEs decreased significantly. In contrast, the mean particle size of APEs did not change significantly when endured thermal treatment from 60 °C to 100 °C, although serious droplet aggregation of smaller droplets was observed in APEs after 100 °C treating.

Upon heating, the strength of hydrophobic interactions is promoted, leading to a more compact micellar structure formation, which will contribute to high thermal stability [37]. As expected, APEs exhibited higher Asta retention than the bare Asta extracts under the same heating conditions (*p* < 0.05) (Figure 4C), because Asta was embedded in hydrophobic zein particles.

In addition, APEs demonstrated higher antioxidant activity on scavenging DPPH and hydroxyl radicals as compared to the bare Asta (*p* < 0.05). It was noted that the heated APEs at 50 °C to 100 °C showed a stable ability on scavenging DPPH and hydroxyl radicals (*p* > 0.05) (Figure 4D,E). The aforementioned results proved that the Asta-loaded Pickering emulsion stabilized using zein/SA had an effective structure to alleviate Asta degradation at the experimental temperature.

### 2.5. Effects of pH and Metal Ions on APEs Stability

#### 2.5.1. Visual Appearance

**Figure 5:**
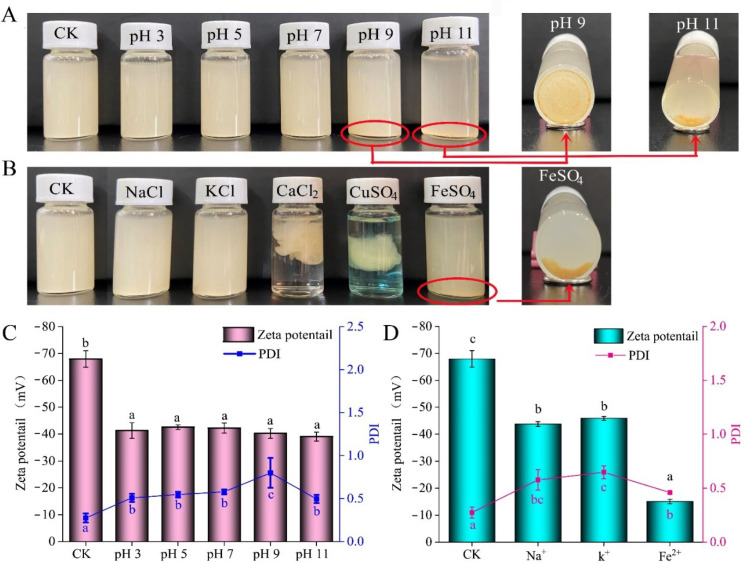
Changes of visual appearance, zeta potential and PDI of APEs after treated at pH 3, 5, 7, 9 and 11 buffers, or 100 mM of Na^+^, K^+^, Ca^2+^, Cu^2+^, and Fe^2+^ metal ions at room temperature. (**A**) Visual appearance after blended with different pH buffers; (**B**) visual appearance after blended with Na^+^, K^+^, Ca^2+^, Cu^2+^, and Fe^2+^, respectively; (**C**) zeta-potential and PDI changes at different pH values (3–11); and (**D**) zeta-potential and PDI changes at Na^+^, K^+^, and Fe^2+^ conditions. All data in (**C**,**D**) were expressed as the mean ± standard deviation (*n* = 3). Different lower-case letters on the bars suggested significant differences (*p* < 0.05).

pH and ionic strength played important roles on the solubility and stability of Pickering emulsions stabilized using polysaccharides and proteins, because the generation of an oil-in-water droplet network in Pickering emulsions relied on the repulsive forces (electrostatic and steric) of polysaccharide–proteins [38,39]. In the current study, after being incubated under different pH values (3–11) or cations (100 mM, Na^+^, K^+^, Ca^2+^, Cu^2+^, and Fe^2+^), APEs demonstrated a different visual appearance (Figure 5A,B). When the pH values ranged from 3.0 to 7.0, no visual changes were observed for APEs. Once the pH of emulsions increased to 9.0, the upper APEs became clear gradually, droplets accumulated, and finally led to phase separation (insert photo in Figure 5A). A similar phenomenon was found at pH 11.0. Our findings proved the pH-dependent property of Asta-loaded Pickering emulsions using zein and SA complex as stabilizers.

As for the coexisting stability with metal ions, APEs remained a uniform dispersion in either Na^+^ or K^+^ conditions. Alkali metal ions have hydration, which can reduce the electrostatic interaction between alkali metal ions and the surface of latex particles. Therefore, the aggregation degree of latex particles is reduced, resulting in higher stability [40]. However, semi-solid emulsion gels were formed after Ca^2+^ or Cu^2+^ addition (Figure 5B). In the presence of Ca^2+^ ions, alginate solutions can form gels by cooperative interaction between Ca^2+^ with blocks of guluronic units (G-blocks) of SA to form ionic bridges between different chains [41]. The most popular model to account for the chain-to-chain association of SA with Ca^2+^ is the “egg box model”. In this model, the G-blocks of SA can form three-dimensional arranged cavities, in which Ca^2+^ ions could crosslink the anionic alginate, like eggs in cardboard egg boxes, and finally fabricate an anionic biopolymer network [42]. Analogous egg-box model was also reported for Cu^2+^ induced alginate gelation [43]. The affinity of divalent ions to SA chains decreased in the following order: Pb > Cu > Cd > Ba > Sr > Ca > Co, Ni, Zn > Mn [44]. Similar to our results, double-cross-linked emulsion gels with dense network microstructures and high viscoelasticity were obtained by Ca^2+^ to crosslink zein and SA [16]. The binding of Ca^2+^ to zein-propylene glycol alginate resulted in a more compact alginate gel [18]. Compared with the same level of Ca^2+^ or Cu^2+^, the binding capacity of Fe^2+^ to SA molecules is weak, and it is not easy to form an integral gel network [45,46]. The aggregates of APEs observed after incubation with Fe^2+^ ions indicated increases of droplet–droplet interaction, which might be ascribed to the total number of anionic groups in SA chains dramatically decreasing in the presence of Fe^2+^ ions. As a result, some zein-coated oil droplets were released from SA network layer and further coalesced through hydrophobic interaction.

#### 2.5.2. Zeta-Potential and PDI

To investigate the stability mechanism of APEs under different pH values and metal ions, the changes of zeta-potential and PDI of APEs were further determined in Figure 5C,D. The initial zeta-potential of APEs was strongly negative (−67.9 ± 3.1 mV), which can be ascribed to formation of negative network structure on the surface of zein coated oil droplets by high negatively charged alginate molecules [16]. After incubation under different pH conditions ranged from 3.0 to 7.0, the zeta-potential of APEs were significantly decreased as compared to that of control (CK) (initial APEs), as well as increased PDI were detected in APEs (*p* < 0.05). Zein particles were to be expected to highly positive charged at pH ranged from 3.0 to 5.0 due to the isoelectric point of zein is around pH 6.2 [47]. Increase of anionic SA adsorption onto the surface of cationic zein-coated droplet could reduce the overall number of anionic groups in the mixed emulsions, thereby leading to a zeta-potential increase. Furthermore, a greater degree of the SA network might result from further crosslinking with cationic zein-coated droplets, which could be an important reason for the increased PDIs.

Under high pH values, such as 9.0 and 11.0, the alkaline conditions had certain neutralization effects on the acidity of the mixed system, and therefore increased the total zeta-potential of APEs. Furthermore, when the pH was above the zein *pI*, the alginate and zein molecules were negatively charged. Consequently, the ability of SA layer to wrap zein particles decreased, resulting in the release of some zein particles from the SA network structure and further aggregation through hydrophobicity (insert photos in Figure 5A). Compared with pH 9.0, the small difference of particles’ distribution (peak 1 and peak 2 in Figure S1) at pH 11.0 could be responsible for its overall decreased PDI (Figure 5C).

In the presence of Na^+^, K^+^ and Fe^2+^, the increase of zeta-potential detected in APEs could be attributed to the increased interaction between cationic metal ions and anionic SA, thereby reduced the total negative zeta-potential of APEs (Figure 5D). However, the strong net negative charge of APEs system under Na^+^, K^+^ and Fe^2+^ ions also suggested that the overall number of anionic groups in the APEs system was greater than cationic zein-coated droplets. This meant that zein particles still entrapped in the network structure of SA, which could account for the stability of APEs in Na^+^ or K^+^ ions. In contrast, a dramatic increase of zeta-potential at Fe^2+^ ions reflected decreases of overall anionic groups in APEs (*p* < 0.05). Therefore, the electrostatic interaction between the positive charge region of zein and negatively charged SA was weakened, and finally resulted in droplets precipitation (insert photos in Figure 5B). In addition, smaller particle size distribution in APEs under Fe^2+^ conditions (Figure S1) might be attributed to smaller polymers formation by Fe^2+^ and negatively charged SA molecules.

#### 2.5.3. Asta Retention Rate

**Figure 6:**
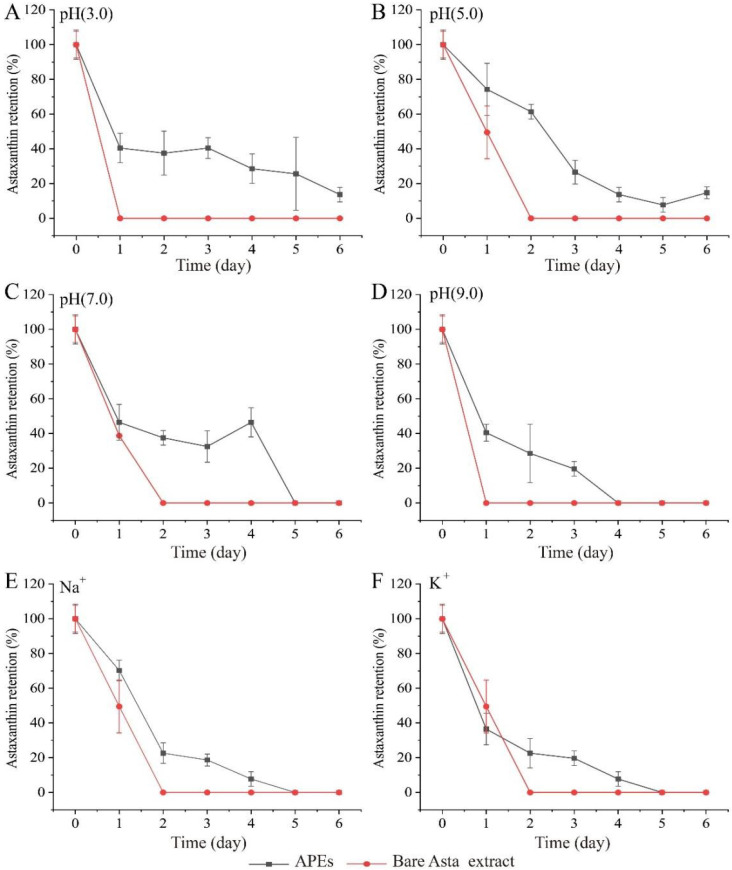
Effects of pH and metal ions on the total Asta retention of APEs during storage for six days under ambient temperature. The bare Asta extract was used as comparison. (**A**) Under pH 3.0 buffer, (**B**) under pH 5.0 buffer, (**C**) under pH 7.0 buffer, (**D**) under pH 9.0 buffer, (**E**) treated with 100 mM of Na^+^, and (**F**) treated with 100 mM of K^+^. Data were expressed as the mean ± standard deviation (*n* = 3).

To evaluate the storage stability of APEs at pH 3.0 to 9.0, Na^+^ and K^+^ conditions (ambient temperature of 25 °C), the total Asta retention of APEs was further measured in Figure 6.

Compared to the bare Asta extract, APEs had higher total Asta retention rate, though the Asta degradation of APEs increased with incubation time increases. Furthermore, APEs showed different properties for Asta degradation under the tested pH values (Figure 6A–D). Under acidic conditions, such as pH 3.0 and pH 5.0, similar Asta retention rates were measured in APEs during storage for up to 6 days. However, at pH 3.0, the Asta retention rate of APEs remained relatively stable from day 1 to day 3, and then gradually decreased after three days of storage. In contrast, a dramatic decrease of Asta retention was found for APEs at pH 5.0 during 4-day storage. At pH 7.0, Asta remained relatively stable from day 1 to day 4 (32% to 46%), and then began to degrade sharply on day 5. By comparison, a dramatic decrease of Asta retention was observed under pH 9.0 condition.

The disassociation of -COOH groups of SA responded to pH change will determine its emulsifying ability [48]. In this study, at acidic conditions above the zein *pI* 6.2 [16], the droplet charges of APEs at pH 3.0 and 5.0 were negative (Figure 5C), suggesting that anionic alginate was adsorbed to the surface of the positive charged zein-stabilized droplets through electrostatic attraction. At alkaline condition, the deprotonated form of SA determined its anionic property, and zein particles were also negatively charged. It indicated that there was less complexation between SA molecules and zein-coated droplets due to strong electrostatic repulsion, and a correspondingly negative charge of total APEs detected at pH 9.0 (Figure 5C). The aggregation of a part of SA also occurred in low pH aqueous solutions, which may reduce the number of SA molecules available for stabilization of emulsion droplets, thus decreasing the emulsion stability [49]. In addition, the pH condition closed to the isoelectric point of zein (*pI* = 6.2) could induce zein particles aggregation to a certain extent, as well as zein coated droplets aggregated at alkaline condition through hydrophobicity. Obviously, the aggregation of zein coated particles was not conducive to the stability of Asta during APEs storage.

Similarly, Asta retention decreased significantly when stored under 100 mM Na^+^ or K^+^ conditions (Figure 6E,F). The interaction between positively charged Na^+^ or K^+^ ions and negatively charged SA reduced the number of SA molecules used to form an external SA network structure to wrap zein coated droplets. Our results indicated that Asta loaded zein particles lack of SA network protection or without strong enough SA interfacial layer could be the main reason for the reduction of Asta retention under the test pH and Na^+^ and K^+^ conditions. Encapsulation of labile Asta into food grade polymeric matrix is considered as a proper approach to provide protection against extreme environment, such as juice and milk [50,51]. In this study, Asta-loaded zein/SA Pickering emulsions were able to provide great protection again pH-induced degradation, with 40% or higher Asta remaining after two days of pH exposure to pH 3.0~7.0, especially around 60% of Asta remained at pH 5.0. A similar result was reported by Zhang et al. (2015), who found the carotenoids droplets stabilized by alginate–soy protein isolate were relatively stable to the droplets aggregation from pH 3 to 7 [48].

#### 2.5.4. Asta Degradation Kinetics

**Figure 7:**
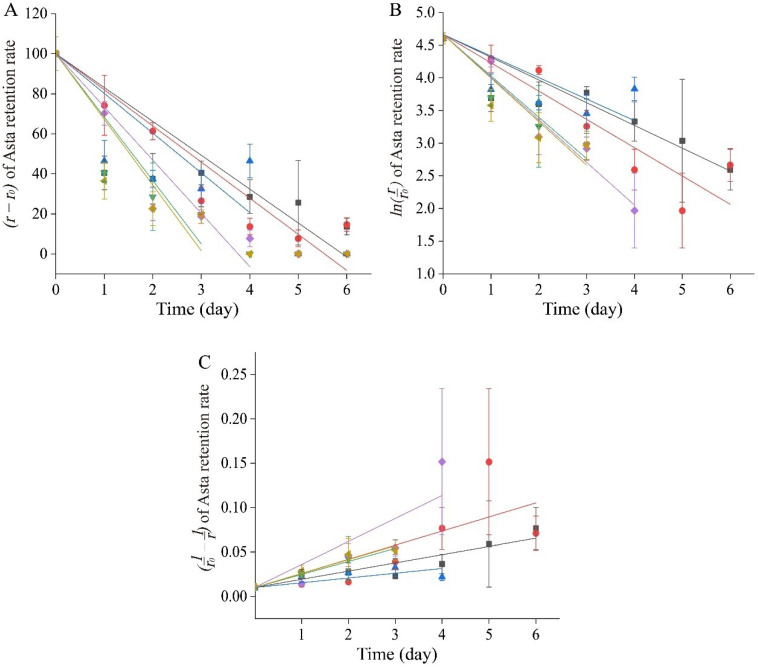
Degradation kinetics of Asta in APEs under conditions of pH 3.0 (■), pH 5.0 (●), pH 7.0 (▲), pH 9.0 (▼), Na^+^ (◆), and K^+^ (◄), respectively. (**A**) Zero order kinetics; (**B**) first order kinetics; and (**C**) second order kinetics. All data were expressed as the mean ± standard deviation (*n* = 3).

The degradation kinetics of encapsulated Asta in APEs under pH 3.0~9.0, Na^+^ and K^+^ conditions was described in Figure 7, and the parameters of *k* and *R*^2^, as well as *t*_1/2_ under the first order kinetics, were summarized in Table 2.

According to the *R*^2^ values, Asta degradation of APEs fit well to the first order kinetics as compared to the zero order or second order kinetics under the tested conditions (Table 2). Similar to our results, the degradation of Asta in microencapsulated flaxseed oil containing crawfish Asta powder followed first-order reaction kinetics [52]. Additionally, Niamnuy, Devahastin, Soponronnarit and Raghavan (2008) found the degradation of dried shrimp Asta fit to the first-order kinetic reaction during storage (4, 15, 25 °C) [53]. During degradation, a higher *k* value relates with higher reaction rate. In this study, the lower *k* values at pH 3.0~7.0 (0.318–0.419) than those at pH 9.0, Na^+^ and K^+^ conditions (>0.60) indicated relatively higher stability of APEs under acidic or neutral environments.

By comparing the *t_1/2_*, it could be seen that the retention rate of Asta at pH 3.0 (2.070 ± 0.059 day) or pH 7.0 (2.200 ± 0.286 day) was more than three times higher as compared to that at pH 9.0 (0.681 ± 0.033 day). Under extreme alkalis conditions, the *cis/trans* isomerization of Asta double bonds, functional group changes and de-esterification [54] could be a contributor for decrease of Asta retention at pH 9.0. Higher stability Asta was reported in an acidic food matrix such as orange juice [50], which was in agreement with Asta oleoresin from *P. rhodozyma* showed the highest stability at pH 4 [55]. Our results suggested potential application prospects of APEs used as fortified additives in acidic and neutral foods.

## 3. Materials and Methods

### 3.1. Materials

Freeze-dried Asta extracts (30.94% of free Asta and 69.06% of Asta esters) derived from Penaeus sinensis by-products were prepared following a protocol performed in our laboratory [20,56]. Free Asta standard (≥98%, HPLC) was obtained from Aladdin Industrial Corporation (Shanghai, China). Zein powder (CAS: 9010-66-6) was purchased from Sigma-Aldrich, Co., USA. Sodium alginate (SA) (CAS: 9005-38-3) was obtained from Sinopharm Chemical Reagent Co., Ltd. (Shanghai, China). All other reagents were of analytical grade and were commercially available.

### 3.2. Preparation of APEs

APEs were prepared referenced a facile anti-solvent procedure with further modifications [12]. Briefly, zein powder was dissolved in 65% of ethanol at ambient temperature, and stirred magnetically for 1 h to form 1 wt% of zein stock solution. Freeze-dried Asta extracts were dissolved in 65% of ethanol solution to reach Asta concentration of 2 μg/mL. Then, the Asta solution was added drop wise into zein solution at a ratio of 1:1 (*v*/*v*) under stirring. After uniform blending, the mixture was evaporated under vacuum (40 °C) to remove off ethanol completely, followed by deionized water addition to restore original volume, thus fabricating Asta loaded zein colloid particles. The Asta-loaded zein colloid was mixed with 2 wt% SA solution at a ratio of 1:1 (*v*/*v*). After completely stirring, the pH of mixture was adjusted to 4.0 using 5 wt% of citric acid solution, homogenized for 2 min under 12,000 rpm, and then kept at 4 °C for 36 h in dark. The generated sediments were collected and lyophilized (LGJ-10 freeze dryer, Brother Instrument Co., Ltd., Zhengzhou, China). The freeze-dried APEs were dissolved in deionized water at 10:1 (*m*/*v*) for further analysis. Meanwhile, the Pickering emulsion without Asta loading was prepared under the same conditions, named as PEs.

### 3.3. Asta Encapsulation Efficiency

The encapsulation efficiency (EE) of Asta in APEs was defined as the total amount of Asta as compared to the amount of surface Asta determined, and expressed in a percentage.

(1) $$\mathrm{EE}\;(\%)=\frac{\mathrm{Total}\;\mathrm{Asta}\;\mathrm{content}\;–\;\mathrm{surface}\;\mathrm{Asta}\;\mathrm{content}}{\mathrm{Total}\;\mathrm{Asta}\;\mathrm{content}}\times 100$$

Total Asta of APEs was extracted as follows: 20 mg of freeze-dried APEs were blended with 2 mL of organic solvent (dichloromethane: ethanol = 3:10, *v*/*v*), treated under sonication (Sb25-12tds ultrasonic cleaning machine, Ningbo Xinzhi Biotechnology Co., Ltd., Ningbo, China) in ice-water bath for 20 min, and centrifuged at 3000*× g* for 5 min at 4 °C (CF-16RN, Hitachi, Japan). The supernatant was collected and measured the absorbance at 477 nm by a 1510 micro-plate reader (Thermo Fisher Scientific Oy, Vantaa, Finland). For surface Asta extraction, the freeze-dried APEs powders were directly vortexed with the organic solvent for 1 min instead of sonication treatment. A calibration curve of Asta was obtained by measuring a series Asta standard concentration ranging from 0 to 10 μg/mL at 477 nm (R^2^ = 0.9926). The Asta content of extraction was calculated as described in Equation (2).

(2) $$\mathrm{Asta}\;\mathrm{content}\;(\mu g/\mathrm{mg})=\frac{c\;\times v}{m}$$

where c was the Asta concentration (μg/mL) determined from the calibration curve of Asta standard; v was the total volume of extract (mL); and m represented the quantity of freeze-dried APEs (mg).

### 3.4. Asta Retention

Asta stability of APEs under different conditions was expressed as Asta retention rate as calculated in Equation (3).

(3) $$\mathrm{Asta}\;\mathrm{retention}\;\mathrm{rate}\;(\%)=\frac{m_{t}}{m_{0}}\times 100$$

m_0_ and m_t_ represented the initial and final total Asta content (μg) before and after treatments.

### 3.5. Particle Size, Particle Charge and PDI Measurements

All of samples were diluted 30 times with deionized water, and agitated well to prevent multiple scattering effects. Then, the mean particle diameter (z-average), particle charge (zeta-potential) and PDI of samples were determined using Zeta-sizer Nano-ZS90 (Malvern Instruments, Worcestershire, UK) with a detection range from 0.3 nm to 5 μm. All measurements were performed in triplicate at 25 °C.

### 3.6. Morphological Characterization

#### 3.6.1. Appearance and Optical Characteristics

The emulsion was photographed after preparation or treated under different conditions. Emulsion sample (50 μL) was dropped on a clear glass slide with a coverslip and observed under an optical microscope (NiKon E100, Tokyo, Japan) magnified by a 40× objective lens.

#### 3.6.2. SEM and TEM Observation

The microscopic surface properties of freeze-dried APEs and PEs were observed by SEM (JSM-7800F, JEOL, Tokyo, Japan). APEs and PEs were diluted with distilled water. Then, 2 μL of emulsions were dripped on copper net and dried naturally. After negatively stained with 2% phosphotungstic acid for 15 min and remove off extra liquid, the morphology of diluted emulsion was observed under TEM (JEM 1200EX, JEOL, Tokyo, Japan).

#### 3.6.3. CLSM Observation

The microstructure of APEs was observed by confocal laser scanning microscopy (LECIA TCS SP5) referenced with the method of Liang et al. (2020) [19], with slight modifications. In brief, 5 μL of diluted samples were transferred onto glass slides and stained with a mixture of fast green FCF dye (0.1 wt% in distilled water, used for protein staining) and Nile Red dye (0.1 wt% in DMSO, used for oil phase staining) fixed at ratio at 1:1 (*v*/*v*). The stained sample was placed on a concave confocal microscope slide and observed images using a 20× magnification lens at excitation wavelength of 633 nm for fast green FCF and 488 nm for Nile Red, respectively.

### 3.7. Rheological Property

The rheological property of APEs was evaluated according to the method described by Liang et al. (2020) [19] at 25 °C using a dynamic shear rheometer (HR20, Waters, Milford, MA, USA) with a 40 mm diameter parallel plate measurement cell. A thin layer of silicone oil was applied to the outer edge of the samples to prevent water loss during measurement. The apparent viscosity (*η*) and shear stress (*τ*) were recorded as a function of shear rate (*γ*) from 0.1 to 100 (s^−1^). The experimental data of APEs flow curves were analyzed using the Cross model. Additionally, PEs were used for comparison.

(4) $$\mathrm{Cross}\;\mathrm{model}:\;\frac{\eta -\eta_{\infty }}{\eta_{0}-\eta_{\infty }}=\frac{1}{1+{\left(\alpha \gamma \right)}^{n}}$$

where *η*, *η_0_* and *η_∞_* represent the viscosity (Pa·s) at any specific shear rate (*γ*), the zero-shear viscosity, and the infinite shear viscosity, respectively. *α* and *n* represent the characteristic time (s) and the rate index, respectively. For dynamic viscoelastic measurements, the angular frequency (*ω*) was set from 0.1 to 100 rad/s within the linear viscoelastic range of samples. The storage modulus (*G′*) and loss modulus (*G′′*) of samples were continuously determined under 1% fixed strain during the test.

### 3.8. Molecular Interaction

#### 3.8.1. XRD Analysis

XRD patter of freeze-dried APEs was determined using an X-ray diffractometer (Smartlab 9 kW, Rigaku Corporation, Tokyo, Japan) equipped with Cu Kα radiation monochromatic filter (acceleration voltage 45 kV, current 40 mA) in the range of 5–90° at a scanning rate of 0.02°/s. Meanwhile, freeze dried PEs, zein and Asta standard were used for comparisons.

#### 3.8.2. FTIR Assay

Freeze-dried APEs were grinded with dried KBr powders, and recorded on a Nicolet 670 spectrometer (Thermo Fisher Scientific Inc., Waltham, MA, USA) from 400 to 4000 cm^−1^ wavelength at a resolution of 4 cm^−1^ and 32 scans per minute. FTIR spectra of PEs, zein and Asta were used for comparisons.

### 3.9. Thermal Stability

The solution of APEs (10 mg/mL in distilled water) was heated at 50 °C, 60 °C, 70 °C, 80 °C, 90 °C and 100 °C for 30 min in water bath, respectively. Then, 1 mL of thermal treated APEs were blended with 1.3 mL of organic solvent (dichloromethane: ethanol = 3:10, *v*/*v*) to extract the total Asta as described in Section 3.3. The changes of optical micrograph, average particle size and total Asta retention rate were determined. Bare Asta extracts (without encapsulation) and PEs were used for comparisons.

### 3.10. Antioxidant Activity

After thermal treatment, the antioxidant activity of APEs on scavenging DPPH and hydroxyl radicals were performed referenced our previous study [57]. In DPPH scavenging activity assay, the absorbance of all groups was measured at 517 nm using a 1510 micro-plate reader (Thermo Fisher Scientifific Oy, Vantaa, Finland) after 60 min of reaction at room temperature. The DPPH radical-scavenging activity was determined according to the Equation (5).

(5) $$\mathrm{DPPH}\;\mathrm{radical-scavenging}\;\mathrm{activity}\;(\%)=\frac{A_{c}-\left(A_{s}-A_{b}\right)}{A_{c}}\times 100$$

where A_c_ represented the control group, with 125 μL of 99.5% ethanol mixed with 25 μL of DPPH solution; A_s_ was the sample group, with 75 μL of the sample solution blended with 50 μL of 99.5% ethanol and 25 μL of 0.02% DPPH ethanol solution; A_b_ represented the sample blank, with 75 μL of the sample solution blended with 75 μL of 99.5% ethanol.

The hydroxyl radical-scavenging activity of all groups was measured according to the assay kit and expressed as U/mL.

### 3.11. pH and Metal Ions Stability

APEs’ solutions (10 mg/mL in distilled water) were blended with the same volume of 0.2 mol/L phosphate buffer (pH values of 3, 5, 7, 9 and 11) or the same volume of 100 mM metal ions (NaCl, KCl, CaCl_2_, CuSO_4_, and FeSO_4_), respectively. After blending, the visual appearances of the samples were recorded with photographs. Meanwhile, the zeta potential and PDI of samples after diluted suitable times were determined.

Based on the results of pH and metal ions stability, the APEs samples were further stored under pH 3.0~9.0, and Na^+^ and K^+^ conditions at ambient temperature in darkness. At the time point of 1, 2, 3, 4, 5 and 6 days, 1 mL of sample solution were pipetted out for Asta retention measurement. All experiments were performed in triplicate. The Asta retention of bare Asta extracts was used as comparison.

### 3.12. Asta Degradation Kinetics

Asta degradation in APEs under different pH and metal ions conditions were analyzed using zero-, first- and second order models referenced the method described by Sun, Ma, Ye, Kakuda and Meng (2010) [58].

(6) $$\mathrm{Zero}\;\mathrm{order}:\;r-r_{0}=-kt$$

where *r_0_* was the initial Asta retention rate (%); *r* represented the Asta retention rate at different time *t* (%); *k* represented the constant of degradation rate; *t* represented time (day). A plot of Asta retention rate (*r − r*_0_) versus time was constructed. The slope of the straight line (*k*) and the correlation coefficient (*R*) were obtained from the trend line of the plot.

In first-order model, based on Equations (7) and (8), was obtained by taking logarithms on both sides, and then rearranged into Equation (9). The *k* value and correlation coefficient (*R*) in first order model were determined through a plot of ln (*r/r*_0_) versus *t* in Equation (9).

(7) $$\mathrm{First}\;\mathrm{order}:\;r=r_{0}exp\left(-kt\right)$$

(8) $$lnr=lnr_{0}-kt$$

(9) $$\ln\left(\frac{r}{r_{0}}\right)=-kt\;$$

In second-order model, a plot of $\left(\frac{1}{r_{0}}-\frac{1}{r}\right)$ versus *t* in Equation (10) was constructed to obtain the *k* value and correlation coefficient (*R*).

(10) $$\mathrm{Sec}\mathrm{ond}\;\mathrm{order}:\;\frac{1}{r_{0}}-\frac{1}{r}=-kt$$

### 3.13. Statistical Analysis

Data were expressed as mean ± standard deviation. One-way variance (ANOVA) and Tukey’s test were analyzed using SPSS^®^ software 19.0 (Version 1.1, 2000, IBM Company, Chicago, IL, USA) to present significant differences among the mean values at the *p* ≤ 0.05

## 4. Conclusions

The prepared APEs showed a double cross-linked structure with a SA layer outside zein particles. After heat treatment at 50 °C to 100 °C for 30 min, APEs still maintained high antioxidant activity on scavenging DPPH and hydroxyl radicals. Homogenous emulsions were observed for APEs under pH 3.0 to 7.0, Na^+^ and K^+^ conditions. APEs demonstrated higher Asta retention during storage at pH 3.0 to 7.0, and dramatically degraded under Alkaline or Na^+^ and K^+^ conditions when stored up to 6 days at ambient temperature. The SA network layer outside zein particles should provide an important protective structure for Asta retention of APEs under acidic and neutral conditions. Our results will provide important information for Asta loaded Pickering emulsion stabilized with zein and SA, and suggest potential application of APEs as an antioxidant additive in mild heating foods. However, further studies should be conducted, such as the storage stability of lyophilized APEs, appropriate addition and sensory attributes to foods, and adsorption properties in vivo in actual food products.

## Figures and Tables

**Table 1:** Cross model fitting parameters of APEs and PEs at shear rate from 0.1 to 100 (s^−1^) under ambient temperature.

Sample	*α* (s)	*n*	*η*0 (Pa·s)	*η∞* (Pa·s)	*R* ^2^	Standard Deviation (SD)
PEs	6.757	0.68	0.135	0.036	0.9897	0.00495
APEs	95.744	0.66	0.813	0.039	0.9974	0.00199

**Table 2:** Degradation kinetics parameters of *k* (rate constant) and *R^2^* (determination coefficients), *t*_1/2_ (half-life periods) of Asta in APEs.

	Zero Order Kinetics		First Order Kinetics		Second Order Kinetics		*t*_1/2_^▲^ (day)
	*k* (%·day^−1^)	*R* ^2^	*k* (day^−1^)	*R* ^2^	*k* (%^−1^·day^−1^)	*R* ^2^	
pH 3	16.841 (0.143)	0.813	0.334 (0.009)	0.994	0.009 (0.0003)	0.962	2.070 (0.059)
pH 5	17.983 (0.689)	0.943	0.419 (0.055)	0.990	0.016 (0.0053)	0.828	1.668 (0.216)
pH 7	19.969 (1.196)	0.858	0.318 (0.043)	0.991	0.005 (0.0004)	0.923	2.200 (0.286)
pH 9	31.529 (1.282)	0.918	0.605 (0.101)	0.997	0.015 (0.0007)	0.998	0.681 (0.033)
Na^+^	26.651 (0.697)	0.950	0.633 (0.031)	0.997	0.026 (0.0065)	0.886	1.095 (0.053)
K^+^	33.042 (0.472)	0.885	0.641 (0.020)	0.994	0.016 (0.0003)	0.988	1.080 (0.034)

Data in the brackets were standard deviations (*n* = 3); ^▲^ *t*_1/2_ represent the half-life periods (day) in the first order kinetics.

## Data Availability

All data presented in the manuscript is available upon request.

## Supplementary Materials

The following supporting information can be downloaded at: https://www.mdpi.com/article/10.3390/ijms23169386/s1.
