# Supplementary figures and images for: Property and Stability of Astaxanthin Emulsion Based on Pickering Emulsion Templating with Zein and Sodium Alginate as Stabilizer
Source: Int J Mol Sci. 2022 Aug 20;23(16):9386. doi: 10.3390/ijms23169386 (PMC9408833; doi:10.3390/ijms23169386)

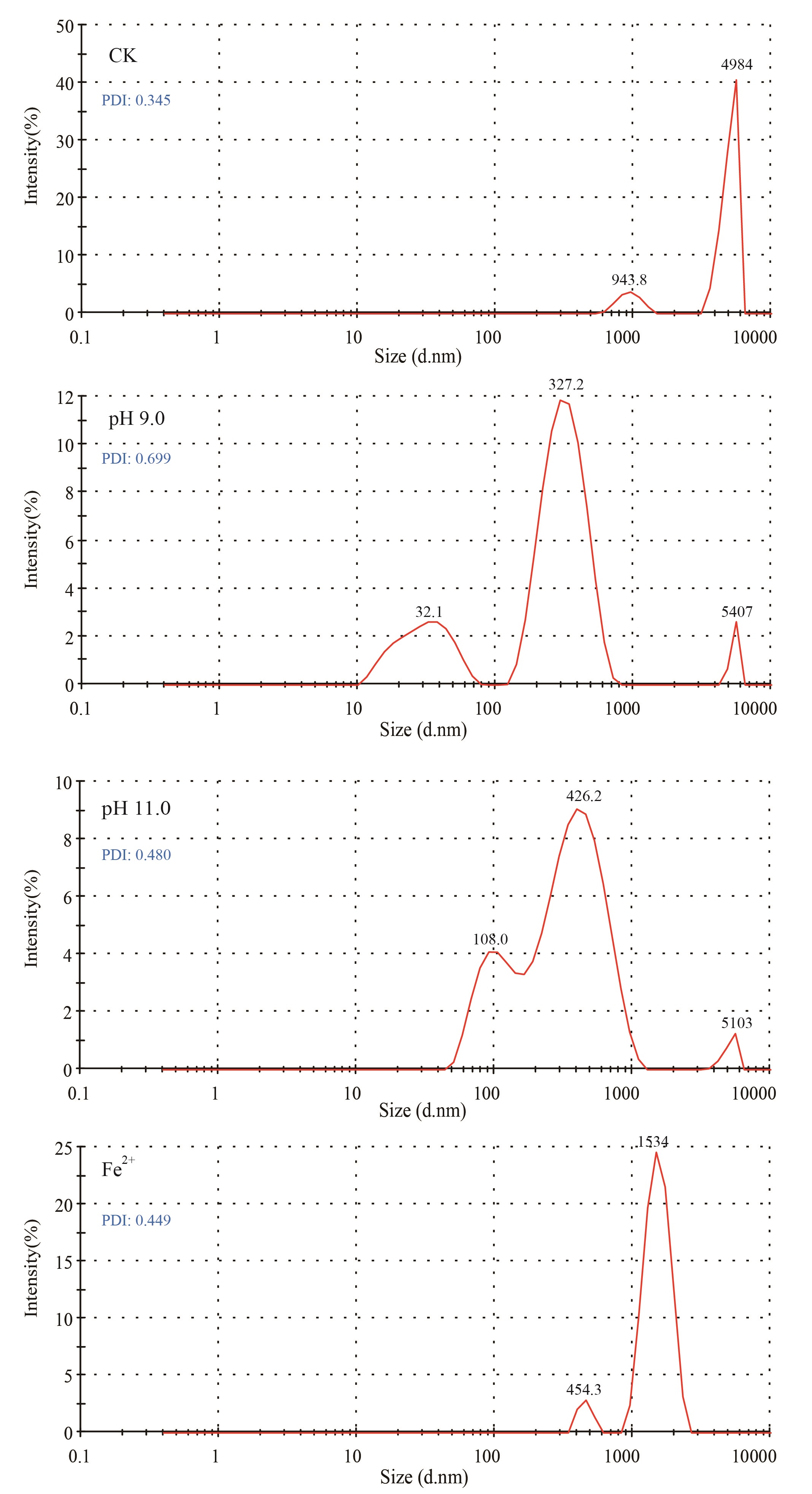

Supplement: Supplementary file 1 [file ijms-23-09386-s001.zip › ijms-1833860-supplementary-Figure S1.png]
